# Abnormal Papillary Muscle Signal on Cine MRI As a Typical Feature of Mitral Valve Prolapse

**DOI:** 10.1038/s41598-020-65983-1

**Published:** 2020-06-08

**Authors:** Alessandra Scatteia, Carmine Emanuele Pascale, Paolo Gallo, Salvatore Pezzullo, Raffaella America, Alberto Maria Cappelletti, Laura Adelaide Dalla Vecchia, Pasquale Guarini, Santo Dellegrottaglie

**Affiliations:** 1 Division of Cardiology “Villa dei Fiori” Hospital, Acerra, Na Italy; 2 Coronary intensive care unit, IRCCS Ospedale San Raffaele, Milan, Italy; 3 IRCCS Istituti Clinici Scientifici Maugeri, Milan, Italy; 4 Zena and Michael A. Wiener Cardiovascular Institute/Marie-Josee and Henry R. Kravis Center for Cardiovascular Health, Icahn School of Medicine at Mount Sinai, New York, NY US

**Keywords:** Anatomy, Cardiology

## Abstract

Background: Mitral valve prolapse (MVP) is characterized by an abnormal movement of the valvular apparatus which may affect the papillary muscles (PMs) function and structure. Aim of the study was to investigate abnormal PM signal in MVP by using cardiac magnetic resonance imaging (MRI). Methods and Results: We enrolled 47 consecutive patients with MVP evaluated by cardiac MRI. Additional groups included healthy volunteers, patients with moderate-to-severe mitral regurgitation (not caused by MVP) and patients with hypertrophic cardiomyopathy. Visual assessment of the PM signals was carried out and the signal intensity (SI) of both the antero-lateral and postero-medial PMs was normalized by that of the left ventricular (LV) parietal myocardium. Our results show that in the MVP group only, the PM signal intensity was significantly lower compared to the one of the LV parietal myocardium. This sign did not correlate with either LV late gadolinium enhancement or positive anamnesis for significant arrhythmias. Conclusions: In MVP patients only, PM signal is significantly reduced compared to LV parietal myocardium (“darker appearance”). The described findings are not clearly related to evidence of myocardial fibrosis, as assessed by MRI, and to previous occurrence of complex ventricular arrhythmias.

## Introduction

Mitral valve prolapse (**MVP**) is the most common valvular disease, with an estimated prevalence of 1% to 3% in the general population^1^. It is characterized by a significant displacement of one or both leaflets into the left atrium, with structural alterations in all components of the valvular leaflets^2^. In patients with MVP, the subvalvular apparatus is also affected and abnormal traction of the papillary muscles (**PMs**) and chordae has been described^3^.

Clinical expression of MVP is extremely variable, but the compendium of potential adverse sequelae is dominated by the occurrence of significant mitral regurgitation^4^. Recent observational studies suggest an association of MVP with sustained ventricular arrhythmias and sudden cardiac death, although the mechanism behind this link is still the object of an active debate^5,6^. Some aspects of the complex pathophysiology of MVP, which adversely affect the tissue composition (with development of myocardial fibrosis) and electrophysiological stability of the PMs and the left ventricular (**LV**) parietal myocardium, may favour the development of a pro-arrhythmic phenotype^7,8^.

Cardiac magnetic resonance imaging (**MRI**) has proven to be an excellent tool for the characterization of MVP^2,9^. Cine MRI allows adequate study of the mitral valvular apparatus, while late gadolinium enhancement (**LGE**) imaging is useful for the detection of areas of PM and LV fibrosis^5,10^.

We hypothesized that in MVP patients, even before fibrosis can be eventually detected with LGE imaging, PMs have an altered signal on cine MRI. In particular, we sought to demonstrate that in MVP the signal intensity (**SI**) of the PMs is typically lower compared to that of LV parietal myocardium as measured on cine images. Furthermore, we looked into a possible association of abnormal PM signal with demonstration of myocardial LGE and occurrence of complex ventricular arrhythmias in MVP patients.

## Methods

### Study subjects

We enrolled a group of consecutive MVP patients who presented to perform a cardiac MRI in order to better characterize the valvular morphology and structure as well as to search for LV myocardial fibrosis (***MVP group***; n = 47). Diagnosis of MVP was based on demonstration by cardiac MRI of >2-mm systolic displacement of any mitral valve leaflet into the left atrium, as measured on a LV long-axis view^9^. In this group of patients, any previous evidence of complex ventricular arrhythmias (defined as repetitive ventricular ectopic beats ≥3 beats in a row) on ECG holter-monitoring, electrophysiological study report or other clinical reports was recorded at the time of the cardiac MRI scan. Patients with history of coronary artery disease, cardiomyopathy, congenital heart defects or cardiac surgery were excluded from the study. Separate cohorts comprised patients with significant (moderate-to-severe) mitral regurgitation from mechanism other than MVP (***MR group***; n = 24), performing a clinical cardiac MRI in order to assess the MR severity or cardiac remodelling, and patients with hypertrophic cardiomyopathy (***HCM group***; n = 15), whose typical indication for cardiac MRI was the quantification of LV hypertrophy (all with unexplained maximal wall thickness ≥15 mm) and LV myocardial fibrosis. Finally, a cohort of healthy volunteers (***HV group***; n = 16) with no known risk factors or history of cardiac disease was recruited for this cardiac MRI study. This study was approved by the Ethics Committee of the “Azienda Sanitaria Locale of Naples, Campania Centro” (N. 333/15-’17 OSS, 5/10/2018), and all subjects (or legal representatives) gave written informed consent. The study was carried out in accordance with relevant guideline and ethical regulations.

### Cardiac MRI

All cardiac MRI studies were performed using a 1.5 Tesla clinical scanner (Philips Achieva; Phillips Healthcare, The Netherlands) and a multi-channel phased-array coil system. The cardiac imaging protocol included breath-hold, ECG-gated steady state free precession (SSFP) cine images acquired in standard 4-chamber, 2-chamber and 3-chamber views as well as a short-axis stack, covering the entire LV from the atrioventricular ring to the apex. Typical acquisition parameters were: slice thickness 8 mm (with 2-mm gap); flip angle 60°; temporal resolution 35–50 ms; repetition time and echo time 4.0/2.0 ms; typical in-plane spatial resolution 1.8 × 1.8 mm. To assess LGE as expression of replacement myocardial fibrosis, post-contrast T1-weighted inversion recovery images were acquired in the same LV short-axis and long-axis planes 10 minutes after 0.1 mmol/kg intravenous administration of gadobutrol (Gadovist, Bayer Healthcare, Berlin, Germany) using an ECG-gated breath-hold 3D sequence (slice thickness 10 mm; flip angle 25°; repetition time and echo time 5.4/2.6 ms; typical in-plance spatial resolution 1.25 × 1.25 mm) and then repeated at 20 minutes applying a 2D magnitude and phase sensitive segmented inversion-recovery sequence (slice thickness 8 mm; flip angle 15°; repetition time and echo time 4.1/2.3; typical in-plane resolution 2 × 2 mm). For both 2D and 3D LGE scans, appropriate inversion times were selected to null LV myocardium.

### Image analysis

All MRI studies were analysed off-line using a commercially available software (CVi_42_, Circle Cardiovascular Imaging Inc., Calgary, Canada). LV volumes were calculated by tracing endocardial contours at end-diastole and end-systole on the LV short-axis stack. Epicardial contours were defined manually at end-systole in each short-axis slice. The LV mass was estimated by multiplying the total myocardial volume for the specific gravity of myocardium as previously described^11,12^. All the values were indexed for the body surface area. The potential presence of LGE areas involving the LV myocardium (including PMs) was visually examined and described.

**Figure 1:**
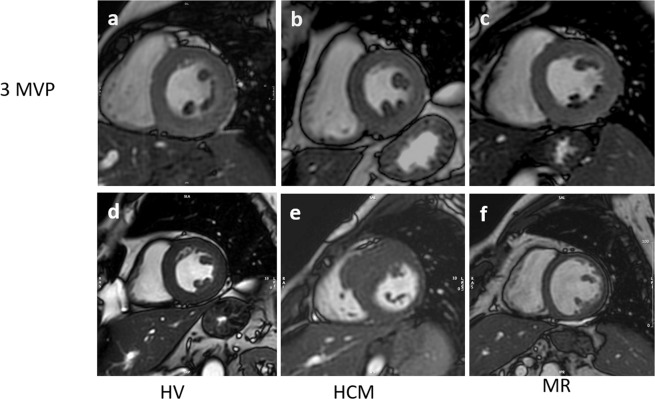
Examples of end-systolic short-axis cine images with clearly lower papillary muscle signal intensity as compared to parietal left ventricular myocardium in MVP patients (**a**–**c**), but not in HV(**d**), HCM (**e**) and MR (**f**).

**Figure 2:**
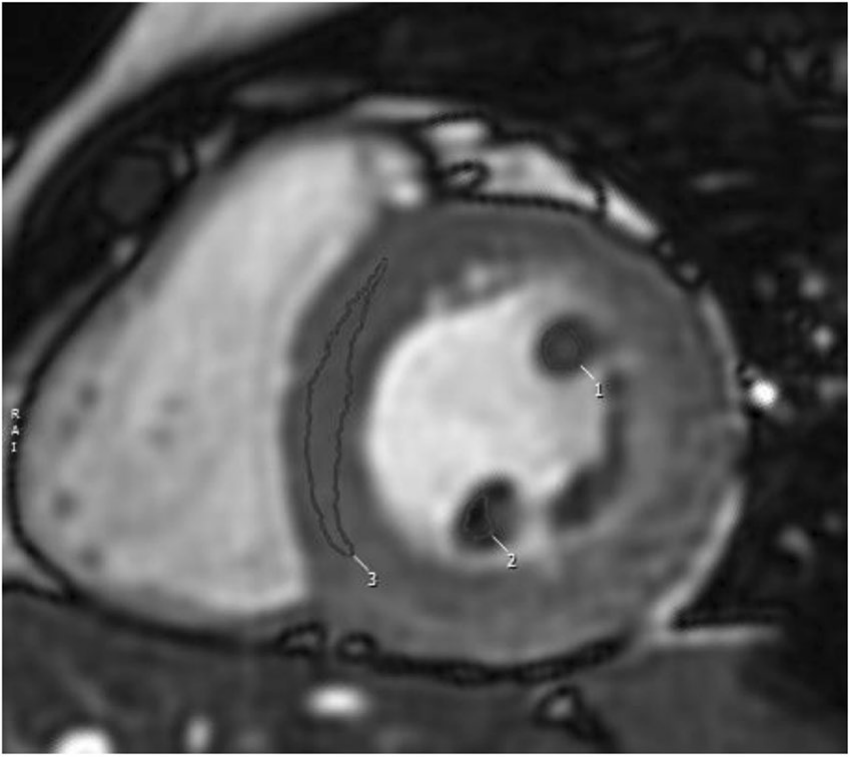
Manually drawing of appropriate region of interest (ROI) on papillary muscle and parietal left ventricle for signal intensity calculation. Post processing with CVi_42_, Circle Cardiovascular Imaging Inc., Calgary, Canada.

For the measurement of PM signal, a mid-ventricular short-axis cine SSFP image in end-systolic phase was selected as it allowed for a better visualization of the PMs. Cine long-axis images were not considered for this analysis as the PMs were not consistently visualized in those images. Initially, the PM signal was visually categorized using a binary score, with “0” being equal to the myocardium SI and “1” representing visually darker PM (Fig. 1). After that, the SI of the antero-lateral and postero-medial PMs was calculated by manually drawing a proper region of interest (**ROI**) and trying to avoid the inclusion of LV cavity signal. Using the same short-axis slice, a different ROI was drawn into the interventricular septum to assess LV parietal SI (Fig. 2). Finally, the antero-lateral and postero-medial PM SIs were indexed for that of LV parietal myocardium obtaining the anterior papillary signal (**APS**) ratio and posterior papillary signal (**PPS**) ratio.

### Reproducibility

Interobserver and intraobserver variabilities for the measurement of APS ratio and PPS ratio were assessed in a subset of 25 cardiac MRI studies randomly selected from the entire study population. For interobserver variability, two experienced readers (A.S. and S.D., both with level III training in cardiac MRI) independently measured APS ratio and PPS ratio without prior knowledge of the clinical data. For intraobserver variability, one reader (A.S.) independently repeated APS ratio and PPS ratio measurements in an identical fashion on two occasions (3 months apart), while blinded to previous results.

### Statistical analysis

Continuous data are summarized as mean ± SD. For data comparison, a paired *t* test or one-way ANOVA was used as appropriate. A χ^2^ test was used to compare categorical variables expressed as proportions. All *p* values are 2 sided and considered statistically significant when <0.05. When required, we provided *p* values after Bonferroni correction^13^. Sensitivity, specificity, positive predictive value (**PPV**), negative predictive value (**NPV**), cut-off values and area under the curve for APS ratio and PPS ratio in identifying MVP patients were derived using receiver-operating characteristics curve (**ROC**) analysis. Cohen’s kappa (*κ*) was used to explore the level of agreement between the visual assessment and the SI measurements. Interobserver and intraobserver reproducibility were assessed by means of intraclass correlation coefficient (**ICC**). All the statistical analyses were performed using IBM SPSS statistics 20.0 software (SPSS Inc, Chicago, IL).

## Results

Baseline characteristics of the included study groups are shown in Table 1. Compared with HV, MVP patients were slightly older, with higher female prevalence and larger LV volumes. Patients in the MR group had increased indexed volumes and reduced LVEF and, as for HCM patients, showed increased LV mass. Bi-leaflets prolapse was observed in the majority (76%) of the MVP population, while isolated anterior or posterior leaflet prolapse was detected in 5% and 19%, respectively.

**Table 1:** Demographic characteristics of the study groups.

	HV (n = 16)	MR (n = 24)	HCM (n = 25)	MVP (n = 47)	*p* value*
Age (years)	29 ± 12	58 ± 12	49 ± 18	42 ± 17	0.01
BSA (cm/kg)	2,04 ± 0,2	1,91 ± 0,2	1,8 ± 0,3	1,78 ± 0,2	0.05
Gender (Female %)	25%	33%	48%	68%	0.05
LVEDV*i* (ml/m²)	72 ± 7	140 ± 52	67 ± 11	92,6 ± 16	<0.001
LVESV*i* (ml/m²)	24 ± 6	95 ± 47	16 ± 7	31,1 ± 9	<0.001
LVmass*i* (gr/m²)	65 ± 10	107 ± 37	110 ± 30	70 ± 15	0,01
LVEF (%)	67 ± 6	37 ± 15	75 ± 7	67 ± 5	0,01
APS ratio	1,06 ± 0,14	1,14 ± 0,16	1,13 ± 0,10	0,70 ± 0,17	<0,001
PPS ratio	0,99 ± 0,15	1,10 ± 0,16	1,03 ± 0,14	0,70 ± 0,20	<0,001

APS ratio: anterior papillary signal ratio; BSA: body surface area; HCM: Hypertrophic cardiomyopathy patients; HV: Healthy volunteers; LVEDVi: index left ventricular end-diastolic volume; LVEF: left ventricular ejection fraction; LVESVi: index left ventricular end-systolic volume; LVmassi: index left ventricular mass; MR: Mitral regurgitation patients; MVP: Mitral valve prolapse patients; PPS ratio: posterior papillary signal ratio.

*p value for between-group differences.

**Figure 3:**
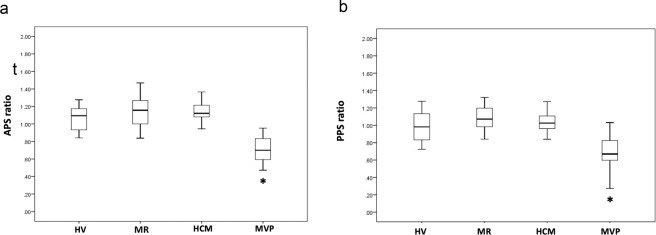
Values of anterior papillary signal (APS) ratio (**a**) and posterior papillary signal (PPS) ratio (**b**) as measured in the different study groups. HCM: Hypertrophic cardiomyopathy group; HV: Healthy volunteers group; MR: Mitral regurgitation group; MVP: Mitral valve prolapse group. Median and interquartile range are indicated. *p value <0,001 between MVP patients and all the other groups.

The APS ratio values were significantly lower in MVP patients (0.70 ± 0.17) than in HV (1.06 ± 0.14), MR patients (1.14 ± 0,16) and HCM patients (1.13 ± 0,10; all groups with *p* < 0.001 vs. MVP; Fig. 3). Similarly, lower PPS ratio values were measured in MVP patients (0.70 ± 0,20) than in HV (0.99 ± 0,15), MR patients (1.10 ± 0,16) and HCM patients (1.03 ± 0,14; all groups with *p* < 0.001 vs. MVP; Fig. 3).

**Figure 4:**
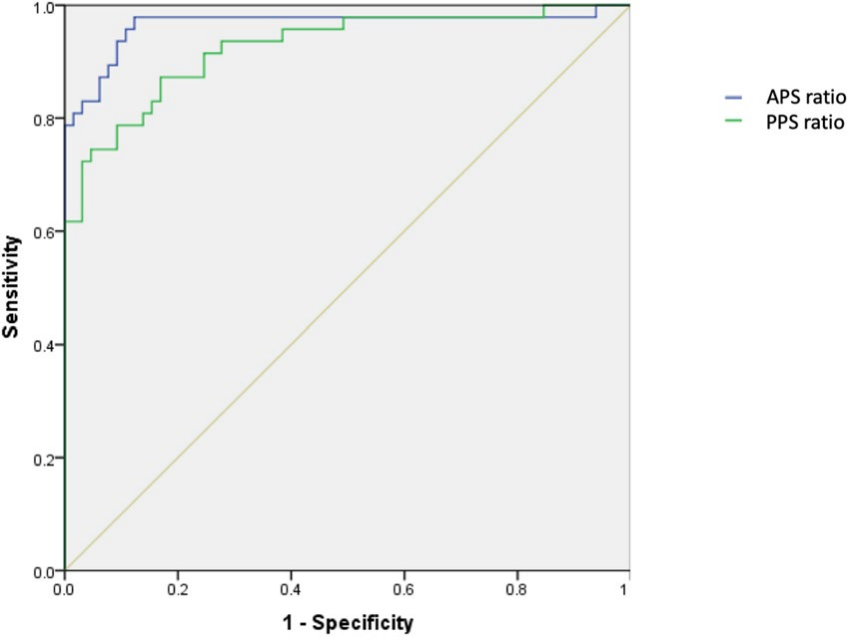
ROC curve analysis for the diagnostic performance of anterior papillary signal (APS) ratio and posterior papillary signal (PPS) ratio in identifying patients with mitral valve prolapse. Area under the curve is 0,96 for APS ratio and 0,92 for PPS ratio.

ROC curve analysis was used to test the diagnostic performance of APS ratio and PPS ratio to identify MVP patients (Fig. 4). A best cut-off value of 0,84 was selected for the APS ratio (81% sensibility, 97% specificity, 95% PPV and 89% NPV) and of 0,80 for the PPS ratio (72% sensibility, 97% specificity, 94% PPV, 82% NPV). The level of agreement between the visual score (darker PM appearance in 37 out of 47 MVP patients) and the indexed SI was good for both the PMs (*κ* = 0,68 for APS ratio and *κ* = 0,62 for PPS ratio).

A total of 10 (20%) patients showed more than mild MR in the MVP group, but no differences were observed in terms of PM signal compared to MVP patients without significant MR (*p* = 0.4). Clear areas of LGE involving the PMs could be identified in 15% (7/47) of the MVP patients. In this group, LV LGE involving the infero-lateral wall was noted (with a non-ischemic pattern) in 1 patient only. Similar values of APS ratio and PPS ratio were measured in the arrhythmic (N = 8, 17%) and non-arrhythmic MVP groups (*p* = 0,4) as well as in MPV patients with or without LGE involving the PMs and/or the LV parietal myocardium (*p* = 0,2).

Overall, SI measurements from cine images showed adequate reproducibility: intraobserver ICCs for APS ratio = 0,90 (95% CI, 0,75-0,96); intraobserver ICCs for PPS ratio = 0,84 (95% CI, 0,70-0,94); interobserver ICC for APS ratio = 0,92 (95% CI, 0,80-0,97); interobserver PPS ratio for PPS ratio = 0,92 (95% CI, 0,79-0,96).

## Discussion

**Figure 5:**
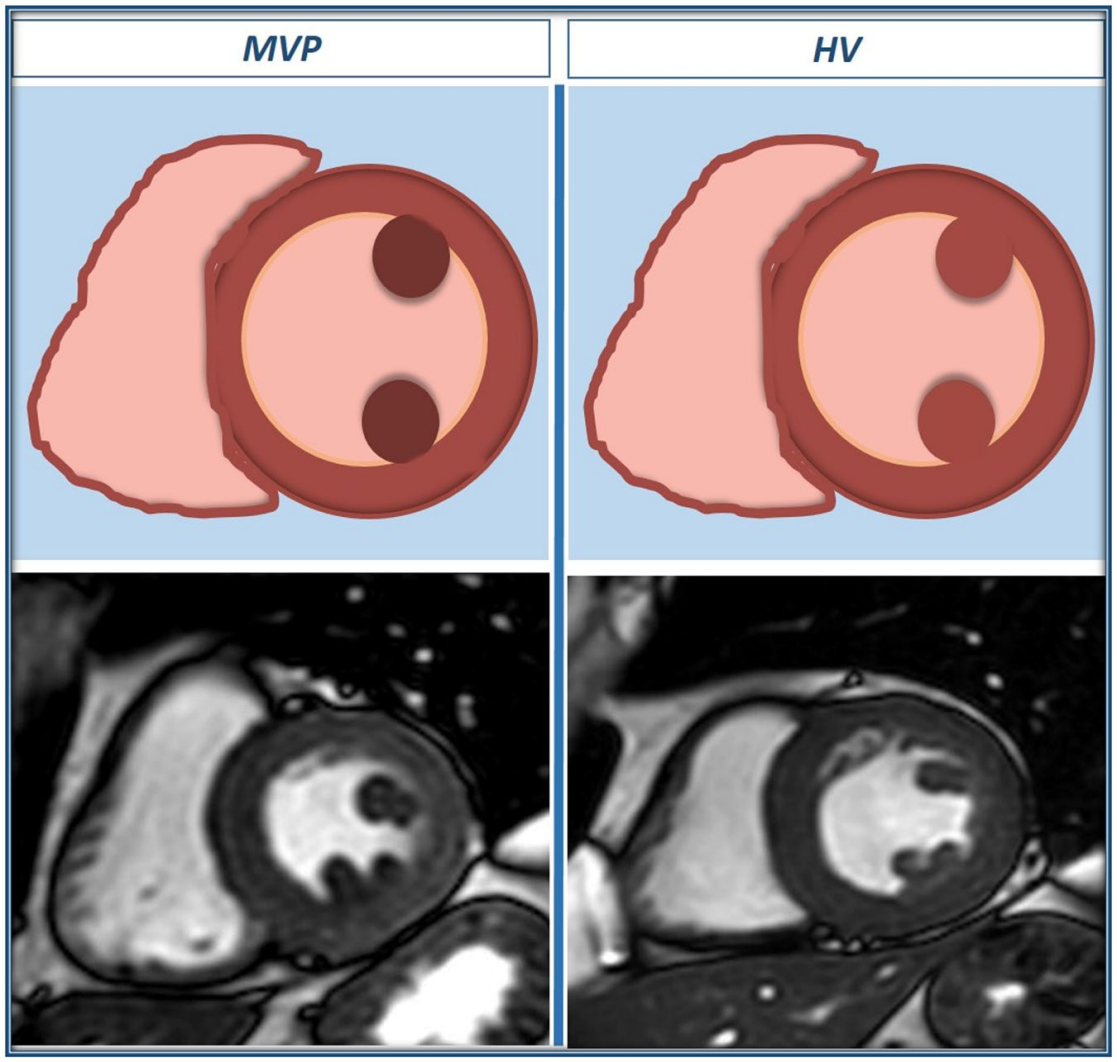
Schematic representation of papillary muscles signal intensity, with correspondent end-systolic short-axis cine images in a MVP case and a HV case.

The main findings of this study are three-fold: (1) on standard cine MRI images obtained in MVP patients, the PM signal is lower compared to LV parietal myocardium (resulting in a typical “darker appearance” of PMs, Fig. 5); (2) this imaging feature is highly specific for MVP, as it is rarely observed in other conditions potentially involving the mitral sub-valvular apparatus; (3) apparently, this signal abnormality involving PMs in patients with MVP is not linked to the demonstration of LV myocardial fibrosis by LGE MRI and to the occurrence of significant ventricular arrhythmias.

### Papillary muscle involvement in valvular and non-valvular conditions

in patients with MVP, redundant mitral leaflets with degenerative changes and elongated chordal apparatus, together with dilated and flattened mitral annulus, create a complex interaction between these structures and the LV. During systole, forces from mitral closure are transmitted to the chordal apparatus and the PMs, causing abnormal traction and excursion^3,10,14–17^. In patients with MVP, Han *et al*. showed that the mitral annulus moves toward the LV apex at peak systole, while the papillary muscles tips tend to move in the opposite direction^18^. Moreover, mitral annular remodelling and deformation has been proven to differ depending on the aetiology of mitral regurgitation. In degenerative mitral regurgitation cases, particularly in those with MVP, mitral annular enlargement associated with a preserved dynamic function has been described^19,20^. Furthermore, mitral annular disjunction is also frequently observed in MVP and could contribute to the typical curling movement of the basal LV segments^21^. Such morphological and mechanical abnormalities might be responsible of a sub-continuous insult to the myocardium and PM stretch, ultimately leading to the development of myocardial hypertrophy and PM or LV fibrosis^17–22^.

In this MRI study and using classical cine SSFP images, we proved that PMs of patients with MVP have different SI compared to the LV parietal myocardium. It can be hypothesized that the described complex mechanical and morphological abnormalities involving the valvular apparatus in MVP might be responsible of the altered PM signal. Interestingly, the PM dark appearance on MRI cine images is evident to the naked eye, with a very good level of agreement between visual assessment and ROI measurements. This implies that this sign may be routinely applied to confirm the MVP diagnosis on cardiac MRI, without the need to add specific images to the acquisition protocol nor to increase the reporting time with post-processing analysis.

Mitral valve abnormalities, such as augmented leaflets lengths, billowing of the leaflets and apical dislocation of papillary muscles, have been previously described in primary or secondary MR as well as in hypertrophic cardiomyopathy^13,23–25^. By assessing the PM signal also in groups of patients with moderate-to-severe MR and with HCM, we found low PM signal to be a highly specific imaging marker of MVP. Indeed, patients in the MR group tend to display higher APS and PPS ratios as compared to controls, but this did not reach statistical significance.

### Relationship with LGE and arrhythmias

previous studies described areas of myocardial fibrosis in patients with MVP, reporting an association with increased risk of arrhythmias or sudden cardiac death^5^. However, the exact incidence of fibrosis involving the LV parietal myocardium and PMs as defined by LGE MRI in MVP patients is still unclear. In about 1/3 of patients with MVP, Kitkungvan *et al*. demonstrated LGE areas in the LV segments (basal inferior and basal inferolateral) adjacent to the postero-medial PM insertion and reported a clear association with sustained ventricular arrhythmias^10^. On the opposite, Han *et al*., by using a highly sensitive MRI sequence, reported LGE involving the PMs in 63% of patients with MVP with no additional LGE areas in the LV parietal myocardium^9^. Basso et. al demonstrated with both autopsy and cardiac MRI high prevalence of PM and LV fibrosis, but this was obtained in a selected group of MVP patients who either died suddenly or presented with complex ventricular arrhythmias^5^. Additional autoptic studies suggested that the presence of LV or PM fibrosis seems to be limited to high risk cases with the presence of extensive bileaflet prolapse and dilatation of the annulus (Barlow disease)^26^. Moreover, when present the fibrosis was typically evident microscopically and not macroscopically and even in cases with MVP-related SCD, a quarter of cases showed histologically normal heart^26,27^.

In our MVP group, LV or PM fibrosis as defined by LGE MRI was detected only in few cases. However, the arrhythmic phenotype in our population of MVP patients was rare, indicating that we may have considered a group of relatively benign cases. Nonetheless, darker PMs were observed to the naked eye in 79% of patients in the MVP group, suggesting that this imaging finding could constitute an early sign of the increased mechanical stress of PMs.

## Limitations

Our cohort of MVP patients was consecutively recruited from a single center and, because of the referral pattern, the evidence of complex ventricular arrhythmias was low. There was no significant difference for both APS and PPS ratios in the arrhythmic and non-arrhythmic MVP sub-groups, but studies performed in MVP populations at higher arrhythmogenic risk could provide additional hints.

Diffuse interstitial fibrosis involving the LV myocardium can be assessed by native or post-contrast T1 mapping techniques^28^. Initial reports described an increased diffuse fibrosis involving the LV parietal myocardium in MVP patients as compared to controls, but reliable T1 measurements are difficult to obtain at the level of PMs^29,30^.

## Conclusions

Our study demonstrates that patients with MVP have a typical abnormal PM signal as compared to LV parietal myocardium on cine cardiac MRI. The finding of a reduced PM signal (“darker appearance”) can accurately differentiate MVP from healthy controls and other conditions potentially involving the mitral sub-valvular apparatus.

This novel imaging marker is not clearly related to the presence of LV and/or PM fibrosis as detected by LGE MRI and to previous occurrence of significant ventricular arrhythmias. More studies are needed to test the diagnostic utility of this sign and its potential role for risk stratification of patients with MVP.
